# Regulatory Functions of L-Carnitine, Acetyl, and Propionyl L-Carnitine in a PCOS Mouse Model: Focus on Antioxidant/Antiglycative Molecular Pathways in the Ovarian Microenvironment

**DOI:** 10.3390/antiox9090867

**Published:** 2020-09-15

**Authors:** Giovanna Di Emidio, Francesco Rea, Martina Placidi, Giulia Rossi, Domenica Cocciolone, Ashraf Virmani, Guido Macchiarelli, Maria Grazia Palmerini, Anna Maria D’Alessandro, Paolo Giovanni Artini, Carla Tatone

**Affiliations:** 1 Department of Life, Health and Environmental Sciences, University of L’Aquila, 67100 L’Aquila, Italy; frea@unite.it (F.R.); martina.placidi@graduate.univaq.it (M.P.); giulia.rossi1@guest.univaq.it (G.R.); domenica.cocciolone@univaq.it (D.C.); guido.macchiarelli@univaq.it (G.M.); mariagrazia.palmerini@univaq.it (M.G.P.); annamaria.dalessandro@univaq.it (A.M.D.); carla.tatone@univaq.it (C.T.); 2 Infertility Service, San Salvatore Hospital, 67100 L’Aquila, Italy; 3 Alfasigma Health Science, 3528 BG Utrecht, The Netherlands; Ashraf.Virmani@alfasigma.com; 4 Department of Obstetrics and Gynecology “P. Fioretti” University of Pisa, 56126 Pisa, Italy; pgartini@gmail.com

**Keywords:** PCOS, L-carnitine (LC), acetyl-L-carnitine (ALC), propionyl-L-carnitine (PLC), mitochondria, advanced glycation end-products, sirtuins, oocyte quality oxidative stress, glycative stress

## Abstract

Polycystic ovary syndrome (PCOS) is a complex metabolic disorder associated with female infertility. Based on energy and antioxidant regulatory functions of carnitines, we investigated whether acyl-L-carnitines improve PCOS phenotype in a mouse model induced by dehydroepiandrosterone (DHEA). CD1 mice received DHEA for 20 days along with two different carnitine formulations: one containing L-carnitine (LC) and acetyl-L-carnitine (ALC), and the other one containing also propionyl-L-carnitine (PLC). We evaluated estrous cyclicity, testosterone level, ovarian follicle health, ovulation rate and oocyte quality, collagen deposition, lipid droplets, and 17ß-HSD IV (17 beta-hydroxysteroid dehydrogenase type IV) expression. Moreover, we analyzed protein expression of SIRT1, SIRT3, SOD2 (superoxide dismutase 2), mitochondrial transcriptional factor A (mtTFA), RAGE (receptor for AGEs), GLO2 (glyoxalase 2) and ovarian accumulation of MG-AGEs (advanced glycation end-products formed by methylglyoxal). Both carnitine formulations ameliorated ovarian PCOS phenotype and positively modulated antioxidant molecular pathways in the ovarian microenvironment. Addition of PLC to LC-ALC formulation mitigated intraovarian MG-AGE accumulation and increased mtTFA expression. In conclusion, our study supports the hypothesis that oral administration of acyl-L-carnitines alleviates ovarian dysfunctions associated with this syndrome and that co-administration of PLC provides better activity. Molecular mechanisms underlying these effects include anti-oxidant/glycative activity and potentiation of mitochondria.

## 1. Introduction

The polycystic ovarian syndrome (PCOS) is a metabolic and endocrine condition affecting 4–21% of women in reproductive age [1,2]. Classic PCOS symptoms include hyperandrogenism, oligo-ovulation, and polycystic ovaries [3]. Moreover, this syndrome is usually associated with insulin resistance, metabolic disorders, and infertility [3], and in the long run with diabetes and cardiovascular disease. Despite PCOS being widespread, the molecular mechanisms leading to its establishment are unclear, thus limiting therapeutic strategies. Recently, many studies have suggested a central role for oxidative and glycative stresses in the pathogenesis of the PCOS and a decreased antioxidant capacity in patients with PCOS [4,5,6,7]. In addition, it has been demonstrated that PCOS patients consuming antioxidants present an amelioration of ovarian function and morphology, with a reduction of oxidative stress [8].

In search for effective antioxidants to employ as a complementary therapeutic approach to improve the prognosis of PCOS patients, L-carnitine (LC) has emerged [9,10]. LC is as an essential cofactor that can be synthesized endogenously or obtained from dietary sources and plays a crucial role in the regulation of cell metabolism. It is essential in fatty acid metabolism by facilitating the transport of long-chain free fatty acids into the mitochondrial matrix, making them available for beta-oxidation [11]. It is also involved in alpha-ketoacids intra-mitochondrial shuttling and oxidation and modulates the intramitochondrial acyl-CoA/CoA ratio. Moreover, LC shuttles acetyl groups from inside to outside the mitochondrial membrane, thus regulating glucose metabolism and sensing cellular energy level. LC has direct antioxidant properties, protects mitochondrial metabolism, and modulates the activities of ROS-producing enzymes [12,13]. For these reasons, it may contribute to the treatment of PCOS and its complications linked to redox imbalance [14]. Together with LC, the endogenous carnitine pool is formed by the short-chain carnitine esters acetyl-L-carnitine (ALC) and propionyl-L-carnitine (PLC). When exogenously administered, ALC and PLC have a higher bioavailability in comparison to LC. In vitro studies have demonstrated that ALC enhances energy production and protects mitochondria against oxidative stress in cellular models resuming several pathological conditions, e.g., Parkinson’s disease, Alzheimer’s dementia [15,16]. Moreover, oral administration of ALC ameliorates blood biochemical parameters of hypertensive patients with increased cardiovascular risk [17]. PLC seems to provide more therapeutic benefits than LC or ALC, possibly due to its pharmacokinetics. Indeed, PLC traverses both cell and mitochondrial membranes and is rapidly converted into free LC and propionyl-coenzyme A (CoA). In these two forms, PLC leads to an enhancement of ATP generation. Under hypoxic condition, PLC improves the efficiency of the Krebs cycle by providing the propionate, which is rapidly transformed into succinate without energy consumption [18]. In addition, PLC stabilizes biomembranes by affecting their molecular dynamics and turnover of phospholipids and acts as intracellular superoxide scavengers, so improving mitochondrial function and reducing DNA damage [19,20,21]. Clinical studies have demonstrated that PLC exerts protective effects against some systemic alterations associated with insulin resistance [22], but there is no information regarding its application in the treatment of PCOS.

Several reports have highlighted the presence of low serum levels of carnitines in PCOS obese and non-obese women, in association with insulin resistance and hyperandrogenism [9,10,23,24]. Therefore, LC supplementation has been successfully employed in PCOS patients, who, as a consequence, showed improvements in hormonal and metabolic parameters, increased energy consumption, a reduction of lipids and body weight [4,9,23,24,25,26,27,28]. In two different studies, LC administration has improved menstrual cycle regularity, ovulation and pregnancy rates [9,23]. At the ovarian level, Kalhori et al. [29] observed in a PCOS mouse model alterations in ovarian volume and folliculogenesis that were partially prevented by LC administration. Genazzani et al. [30] administered ALC in association with LC in PCOS women, demonstrating its beneficial effects on insulin resistance. Nevertheless, beneficial effects of LC and ALC on ovarian physiology remain unclear, so limiting their therapeutic employment in women with PCOS. Concerning PLC, although it is well-known, its activity as antioxidant and promoter of mitochondrial functions in other organs and pathologies, the application of this carnitine in the treatment of PCOS has never been explored.

Based on the numerous regulatory functions of carnitines and their esters in energy metabolism and mitochondrial function, we investigated possible efficacy of LC-ALC formulation on PCOS ovarian physiology and underlying mechanisms and whether the addition of PLC to LC and ALC would have more beneficial effects on PCOS ovaries. To this end, we employed a PCOS mouse model developed in CD1 strain by dehydroepiandrosterone (DHEA) administration and focused on possible modulation of oxidative and glycative stress previously characterized in this model [7] by different carnitine formulations.

## 2. Materials and Methods

### 2.1. Animals

Outbred CD-1 mice (Charles River Italia s.r.l., Calco, Italy) were maintained in a temperature-controlled environment under a 12 h light/dark cycle (07:00–19:00) and free access to feed and water ad libitum. All the experiments were carried out in in conformity with national and international laws and policies (European Economic Community Council Directive 86/609, OJ 358, 1 12 December 1987; Italian Legislative Decree 116/92, Gazzetta Ufficiale della Repubblica Italiana n. 40, 18 February 1992; National Institutes of Health Guide for the Care and Use of Laboratory Animals, NIH publication no. 85–23, 1985). The project was approved by the Italian Ministry of Health and the internal Committee of the University of L’Aquila (authorization n. 269/2018-PR).

Forty four-week-old, body weight 20–21 g, young CD-1 female mice were randomly assigned to four groups (10 in each). To establish the PCOS model, according to [7], mice were daily injected (sub-cutaneously) with DHEA (6 mg/100 g body weight, 100 μL/mouse in sesame oil with 10% of 95% ethanol, Sigma) for 20 consecutive days (DHEA mice). The vehicle control group was injected with 0.09 mL sesame oil and 0.01 mL 95% ethanol daily for 20 consecutive days (control mice). At the same time, mice received, by oral gavage, the carnitine formulation 1 (0.40 mg LC, 0.20 mg ALC, DHEA + C1) and the carnitine formulation 2 (0.40 mg LC, 0.20 mg ALC, 0.08 mg PLC, DHEA + C2) dissolved in water 100 μL/mouse daily for 20 consecutive days. The control and DHEA groups received daily oral administration of water for 20 consecutive days.

Mice were sacrificed by an inhalant overdose of carbon dioxide (CO_2_, 10–30%), followed by cervical dislocation. All efforts were made to minimize suffering.

### 2.2. Estrous Cycle Determination

Analysis of vaginal smears was performed daily from the seventh day after the first injection of DHEA or vehicle. The stages of the estrous cycle were determined daily based on direct examination (“wet smear”) technique [7]. Vaginal cells were collected via saline lavage and then observed under light microscope with a 10× objective. Predominant nucleated epithelial cells and some cornified epithelial cells indicated the proestrous stage, predominant cornified squamous epithelial cells indicated the estrous stage, both cornified squamous epithelial cells and leukocytes indicated the metaestrous stage, and predominant leukocytes indicated the diestrous stage.

### 2.3. Hormone Assay

At the end of the treatment, five mice per group underwent submandibular facial vein blood collections (survival bleeds) [31]. Then, 100 μL of blood per mouse were collected in a glass micropipette capillary tube. Serum was obtained after blood centrifugation at 3000× *g* for 5 min at room temperature. Testosterone serum concentrations were measured using a mouse ELISA assay (Intra-Assay: CV < 8%, Inter-Assay: CV < 10%; FineTest, Wuhan, China).

### 2.4. Superovulation Induction and Oocyte Collection

In order to obtain mature oocytes, at 48 h after the last treatment, five mice per group were treated for the induction of super ovulation by intraperitoneal injection of 10 IU PMSG (Folligon; Intervet-International, Boxmeer, The Netherlands), followed by 10 IU hCG (Profasi HP 2000; Serono, Roma, Italy) 48 h apart. Fifteen hours after hCG, oviducts were removed, and oocytes were arrested at metaphase II (MII) stage were isolated after a brief exposure to 0.3 mg/mL hyaluronidase (Sigma-Aldrich, St. Louis, MO, USA).

### 2.5. H&E Staining and Ovarian Follicle Classification and Counting

Part of the ovaries was sliced in half. One half of each ovaries was fixed in 3.7% paraformaldehyde (PFA) in PBS (Bio-Optica, Milan, Italy) for 12–16 h haematoxylin and eosin (H&E) staining, dehydrated in the ascending series of alcohol, clarified in xylene and embedded in paraffin blocks. Samples were cut with a microtome (Leica SMR2000, Wetzlar, Germany) and sliced into 6 µm serial sections. Sections were then deparaffined and hydrated through xylenes and descending series of alcohol, stained with H&E according to the manufacture’s instruction (Bio Optica, Milan, Italy) and observed by light microscopy (Zeiss Axiostar Plus, Oberkochen, Germany). Follicle classification and counting was performed by counting at least three serial sections/slide (20 slices in average for ovary), spaced ~50 µm each.

Stained ovarian follicles were classified as normal or degenerated for the qualitative evaluation. The normal follicles were those that presented the complete basal membrane, absence of pyknotic bodies in the oocyte nucleus, without signs of oocyte and/or granular degeneration. Normal follicles were classified according Gougeon’s classification [32] into (i) primordial follicle, oocyte surrounded by a single layer of flattened pre-granulosa cells; (ii) primary follicle, oocyte showing a single layer of cuboidal granulosa cells; (iii) secondary follicle, with at least two complete layers of granulosa cells; and (iv) antral follicle, with development of an antral cavity.

### 2.6. Heidenhain’s AZAN Trichrome Staining

Paraffin embedded sections of formalin-fixed ovarian tissue were deparaffinized and hydrated through xylenes and graded alcohol series and processed for trichrome staining (Electron Microscopy Sciences, Danvers, MA, USA), according to the manufacturer’s instructions.

### 2.7. Ovarian Analysis of Lipid Droplets

Some ovaries were fixed in 3.7% PFA/PBS (Bio Optica, Milan, Italy) for the 4,4-difluoro-1,3,5,7,8-pentamethyl 4-bora-3a,4a-diaza-sindacene (BODIPY 493/503 Molecular Probes, Invitrogen by ThermoFisher Scientific, Waltham, MA, USA) staining. Ovarian sections were incubated with 1 μg/mL BODIPY for 10 min, at RT. Finally, sections were mounted with Vectashield Mounting Medium with Dapi (Vector Laboratories, Burlingame, CA, USA) and examined under a Leica TCS SP5 confocal microscope (Mannheim, Germany).

### 2.8. Ovarian Immunohistochemical Analysis

Paraffin embedded sections of formalin-fixed ovarian tissue were deparaffinized and hydrated through xylenes and graded alcohol series. To increase the immunoreactivity, the sections were boiled in 10 mM citrate buffer (pH, 6.1 Bio-Optica, Milan, Italy) in a microwave at 720 W (3 cycles/3 min each). The sections were then subjected to treatment for blocking endogenous peroxidase activity (Dako). After thorough washing, sections were incubated with M.O.M mouse IgG blocking reagent overnight at 4 °C (Vector Laboratories) according to the manufacturer’s protocol. Then, sections were incubated with mouse monoclonal antibody against methylglyoxal-advanced glycation end-products (MG-AGE) (Arg-Pyrimidine, AGE06B, BioLogo, 1:100) diluted in M.O.M diluent for 30 min, according to the Vector Laboratories instructions. MG-AGE was revealed by biotinylated anti-mouse IgG followed by streptavidin-HRP, DAB substrate buffer, and DAB (Dako kit), according to the manufacturer’s instructions. Counterstaining was performed with hematoxylin (Bio-Optica). Negative controls were performed by omitting primary antibody and substituting it with M.O.M diluent alone. Finally, sections were dehydrated and mounted with Neomount (Merck, Darmstadt, Germany). They were observed and photographed under a Leitz Laborlux S microscope (Germany) equipped with an Olympus digital compact camera.

### 2.9. Western Blot Analysis

Part of the ovaries stored at −80 °C were processed for protein extraction. Ovarian tissues were homogenized in RIPA buffer by repeated freeze/thaw cycles in liquid nitrogen. After centrifugation (14,000 rpm for 90 min at 4 °C), the supernatants were collected for protein analysis. Protein concentration was determined by BCA protein assay kit (Pierce, Rockford, IL, USA). Protein samples were separated by SDS-PAGE and transferred to a polyvinylidene difluoride membrane (Sigma-Aldrich, St. Louis, MO, USA). Non-specific binding sites were blocked for 1 h at room temperature with 5% not fat dry milk (Bio-Rad Laboratories, Segrate, Italy) in Tris-buffered saline containing 0.05% Tween 20 (TBS-T). Membranes were incubated with polyclonal rabbit anti-17 beta-hydroxysteroid dehydrogenase type IV (17ß-HSD IV) antibody (PA5-21522, ThermoFisher Scientific, Waltham, MA, USA, 1:250), anti-sirtuin 1 (SIRT1) antibody (Ab12193, Abcam; 1:700), anti-SIRT3 (Ab86671, Abcam, Cambridge, UK; 1:500), anti-superoxide dismutase 2 (SOD2) antibody (Ab86087, Abcam; 1:1000), anti-receptor for AGE (RAGE) (PA1-075, ThermoFisher Scientific, 1:750), anti-glyoxalase 2 (GLO2) antibody (Ab154108, Abcam; 1:500) or monoclonal mouse anti-mitochondrial transcription factor A (mtTFA) antibody (SC-166965, Santa Cruz, 1:250), anti-glyceraldehyde-3-phosphate dehydrogenase (GAPDH) (TA802519, OriGene Technologies Inc, Rockville, MD, USA, 1:750) overnight at 4 °C, followed by incubation with horseradish peroxidase (HRP) conjugated anti-rabbit (BA1054, Boster Biological Technology Co., Ltd., 1:3000) or anti-mouse secondary antibody (Ab6728, Abcam, 1:2000) for 1 h at room temperature. After washing, specific immunoreactive complexes were detected by ECL kit (Thermo Scientific, Waltham, MA, USA) and Uvitec Cambridge system (Alliance series, Cambridge, UK). The bands were normalized for GAPDH using ImageJ 1.44 p software, and values were given as relative units (RU). All the experiments were performed in triplicate.

### 2.10. Analysis of DNA Distribution and Spindle Configuration of In Vivo Matured MII Oocytes

In vivo matured MII oocytes were fixed for immunofluorescence and labelled by mouse anti-α-tubulin (T9026, Sigma Aldrich, 1:200) primary antibody overnight at 4 °C and secondary goat anti mouse-antibody conjugated with Alexa 594 (A90-137D4, Bethyl Laboratories Inc., 1:500, Montgomery, TX, USA) for 1 h at room temperature. Chromatin staining was performed by 5 μg/mL Hoechst 33,342 (Sigma-Aldrich) for 5 min at room temperature. In negative control oocytes, the primary antibody was omitted. Oocytes were mounted on slides and analyzed under epifluorescence microscope at 100× magnification.

### 2.11. Statistical Analysis

Samples obtained from mice of the same experimental groups were pooled. Experiments were repeated three times. All data are presented as mean ± SEM of at least three replicates. Statistical analysis was assessed by t-test. Analyses were performed using the SigmaStat software (Jandel Scientific Corporation, San Rafael, CA, USA). *p*-values < 0.05 were considered statistically significant.

## 3. Results

### 3.1. Administration of Carnitine Formulations Ameliorated PCOS Phenotype Induced by DHEA

**Figure 1:**
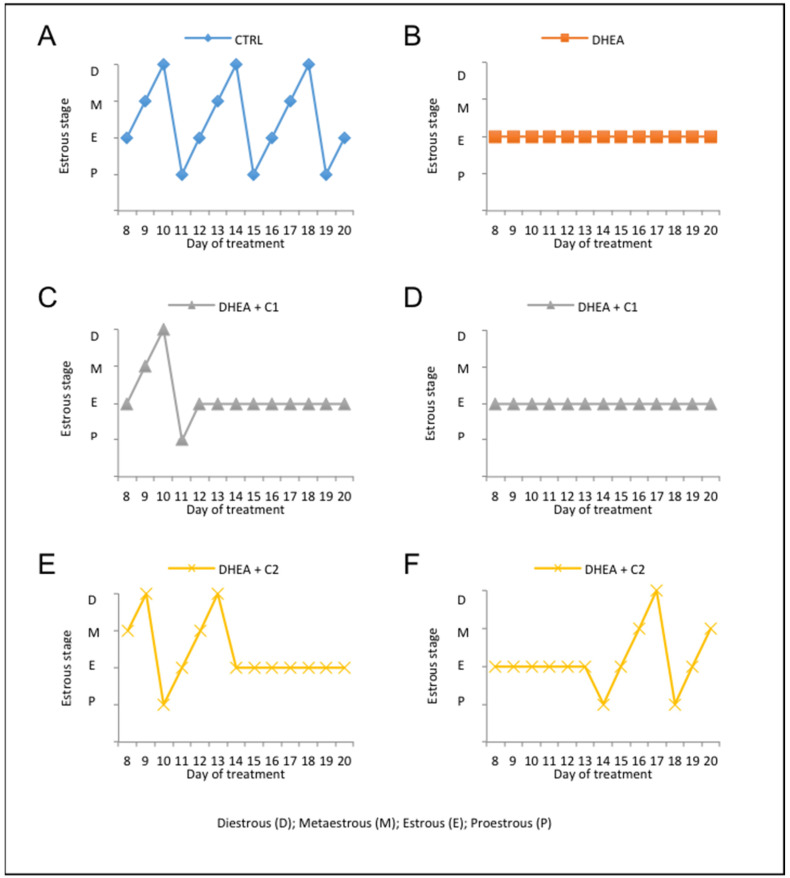
Administration of carnitine formulations ameliorated estrous cyclity disrupted by dehydroepiandrosterone (DHEA). The stages of the estrous cycle, assessed by analysis of vaginal smears, was performed daily from the seventh day after the first injection of DHEA. Representative estrous cycle of one mouse from control (**A**) and DHEA group (**B**). Representative estrous cycle of two mouse from DHEA + C1: 20% of DHEA + C1 mice showed normal estrous during the second week of treatment that was lost in the last week of treatment (**C**); 80% of DHEA + C1 mice did not present estrous during the whole length of treatment (**D**). Representative estrous cycle of two mice from DHEA + C2: 50% DHEA + C2 mice presented normal estrous in the second week of treatment only (**E**); 50% of DHEA + C2 mice showed normal estrous in the final week of treatment (**F**).

To investigate whether administration of carnitine formulations can ameliorate the PCOS phenotype induced by DHEA, we monitored testosterone level, estrous cyclicity, follicle population, and accumulation of collagen and lipid droplets. As previously published [7], DHEA treatment in CD1 mice resulted in the loss of estrous cyclicity, elevated number of atretic follicles, and increased collagen deposition and lipid droplets, which are considered hallmarks of PCOS. After one week of DHEA administration, estrous cycle was monitored daily. As expected, most mice in the control group (90%) showed normal estrous cyclicity (Figure 1A), while DHEA treatment induced a complete loss of estrous cycle (Figure 1B). Administration of carnitines formulation 1 did not prevent loss of estrous cyclicity induced by DHEA in 80% of mice during the whole length of treatment (Figure 1D). During the second week of DHEA treatment about 20% of mice receiving carnitines formulation 1 showed normal estrous, that was lost in the last week of treatment (Figure 1C). By contrast, 50% of mice receiving carnitines formulation 2 presented normal estrous in the second week of treatment only (Figure 1E), while in 50% of mice, the carnitines formulation 2 was able to restore normal estrous in the final week of treatment (Figure 1F).

When we evaluated the serum testosterone, we found undetectable levels in control mice. By contrast, DHEA mice showed a mean value of 0.12 ± 0.05 (S.E.M.) ng/mL. Administration of carnitine formulation 1 during DHEA treatment was not able to prevent testosterone increase, whose serum levels (0.20 ± 0.03 ng/mL) were similar to those observed in the DHEA mice (*p* = 0.13, *t*-test). In contrast, when carnitine formulation 2 was administered to DHEA mice, testosterone decreased to undetectable levels.

**Figure 2:**
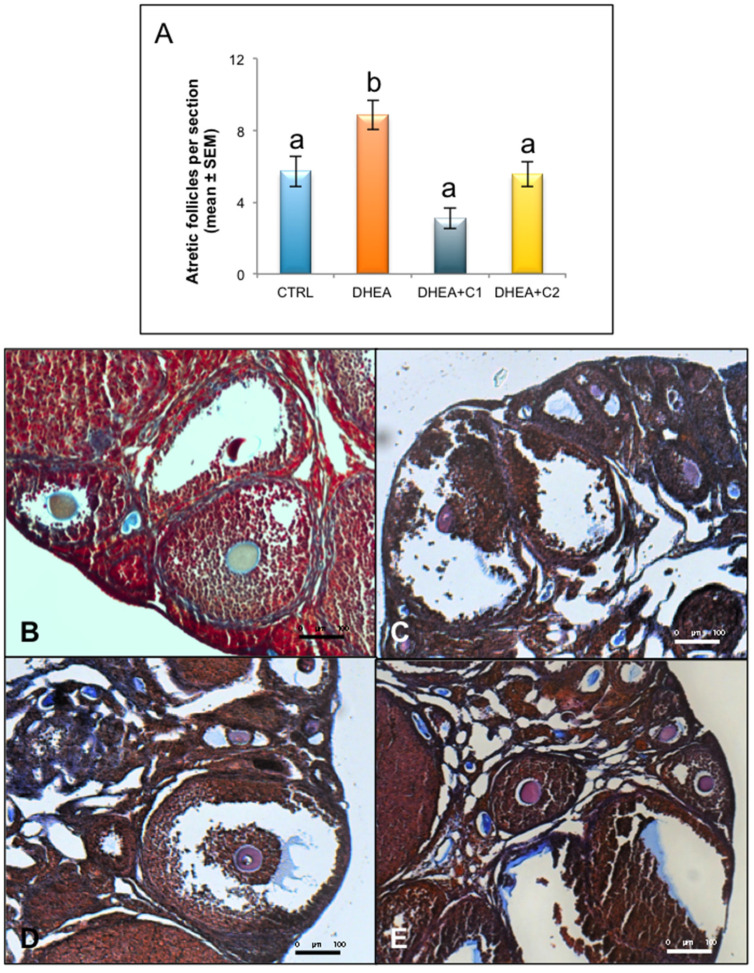
Administration of carnitine formulations ameliorated follicle atresia and collagen deposition induced by DHEA. Part of the ovaries was fixed in 3.7% PFA. After haematoxylin and eosin (H&E) staining, ovarian follicles were scored. (**A**) Atretic follicles observed in different experimental groups are shown. Values are presented as means ± SEM. a, b *p* < 0.05, *t*-test. Part of paraffin embedded sections of formalin-fixed ovarian tissue was processed for trichrome staining, in order to highlight collagen deposition. Representative images of trichrome staining in CTRL (**B**), DHEA (**C**), DHEA + C1 (**D**) and DHEA + C2 (**E**) mice are shown.

As shown in Figure 2A, analysis of the follicle population revealed, as expected, that DHEA treatment induced a highly significant in atretic follicles with respect to controls. Administration of both carnitine formulations was able to prevent this effect.

**Figure 3:**
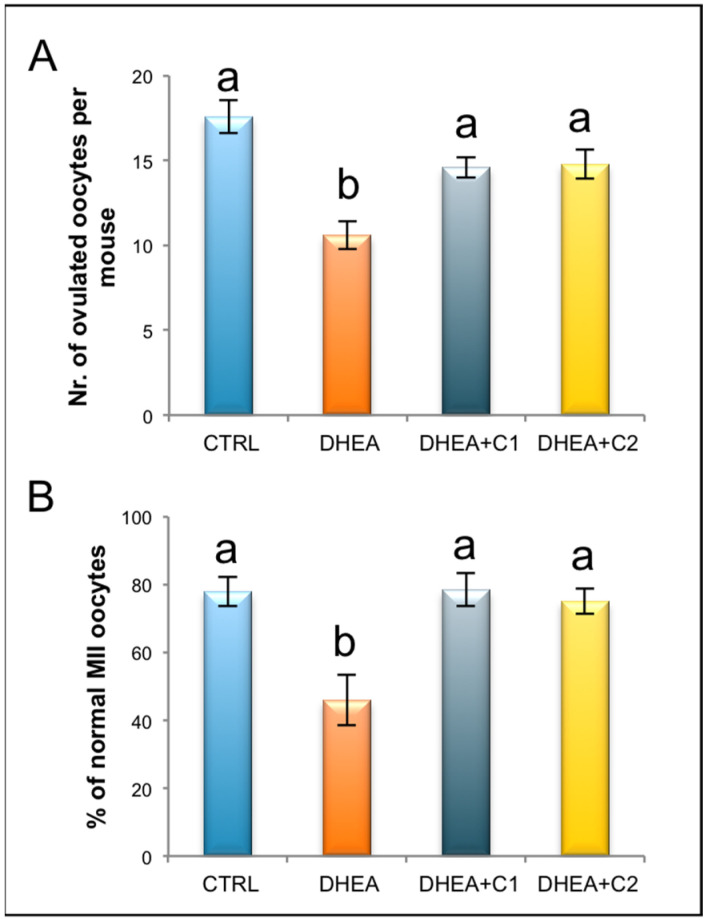
Administration of carnitine formulations improved number and quality of ovulated MII oocytes. Five mice per group were subjected to ovarian stimulation to induce superovulation. (**A**) Mean number of ovulated oocytes per mouse in CTRL, DHEA, DHEA + C1, and DHEA + C2 mice are shown. (**B**) Percentage of normal metaphase II (MII) oocytes in CTRL, DHEA, DHEA + C1, and DHEA + C2 mice are shown. Values are presented as means ± SEM. a, b *p* < 0.05, *t*-test.

After ovarian stimulation with gonadotropins, DHEA-induced PCOS mice exhibited a reduced number of ovulated oocytes per mouse. Administration of both carnitine formulations was effective in increasing the number of ovulated oocytes to the level of control group (Figure 3A).

Then, we focused on the quality of the MII oocytes and more specifically on the presence of a normal metaphase configuration. The MII plate was classified as “normal” when microtubules formed two opposite poles in association with a normal chromosomal distribution or ‘aberrant’ if microtubule structures displayed reduced size of the spindle or lost normal poles or if disorganized microtubule patterns were observed in association with scattered, decondensed or disorganized chromosomes [33]. As shown in Figure 3B, DHEA mice produced a larger number of abnormal oocytes in comparison to control group and both the carnitine formulations were able to counteract this effect as shown by values similar to controls.

DHEA ovaries subjected to trichrome stain were characterized by a fibrotic ovarian cortex (Figure 2B–E). In particular, secondary and antral follicles presented walls with a concentric and network-like collagen distribution, that was more pronounced when compared with ovaries from control mice. Corpora lutea from DHEA ovaries also presented an intense staining of collagen and infiltration of blood cells. When focusing DHEA + C1 ovaries, trichrome staining evidenced a fibrotic aspect, characterized by an abundant presence of intrafollicular and stromal collagen fibers. A reduction of collagen, and therefore of the fibrosis, was found in the DHEA + C2 group, both in the cortex than in the medulla (Figure 2B–E).

**Figure 4:**
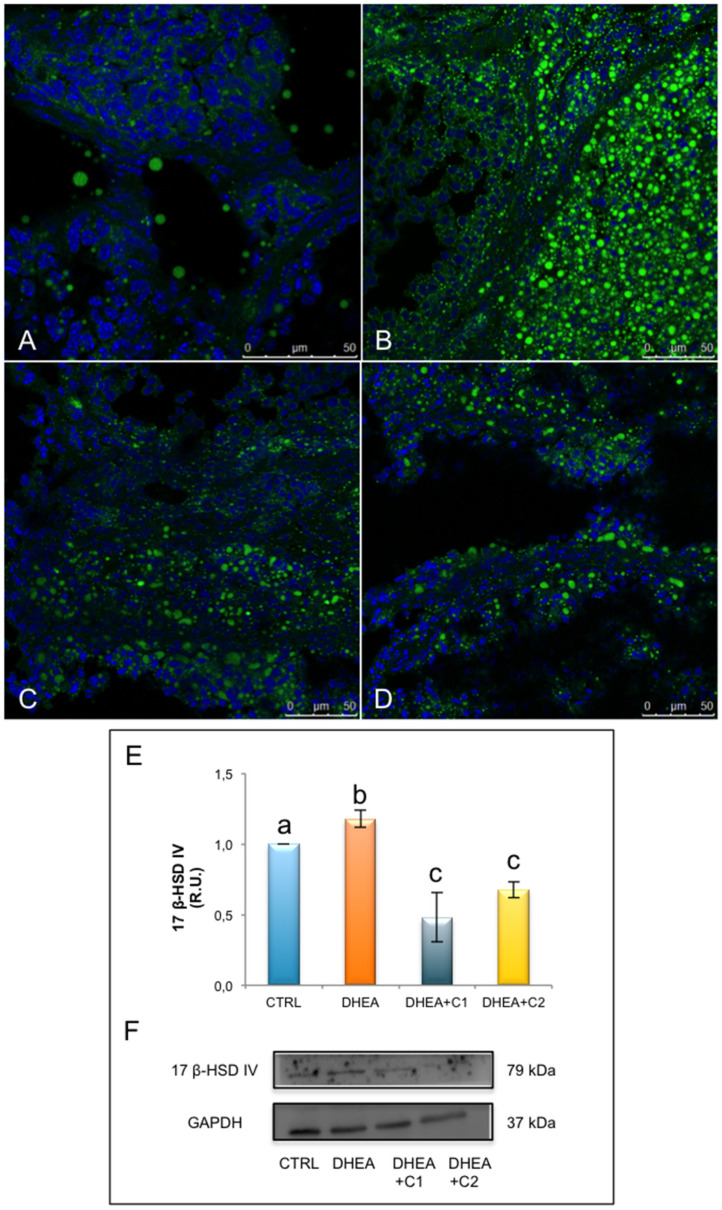
Administration of carnitine formulations reduced lipid content and expression of 17ß-HSD IV, a key enzyme of steroidogenesis. Part of the fixed ovaries were staining by BODIPY 493/503 (green). Representative images of CTRL (**A**), DHEA (**B**), DHEA + C1 (**C**), and DHEA + C2 (**D**) ovaries are shown. DNA is stained by Dapi (blue). Part of the ovaries was processed for protein analysis. Western blot analysis of 17ß-HSD IV (**E**) and representative images (**F**) are shown. Data are presented as means ± SEM of densitometric analysis of immunoreactive bands normalized to internal reference protein (GAPDH). a, b, c *p* < 0.05, *t*-test.

Analysis of BODIPY staining evidenced a stronger signal in DHEA ovaries when compared with controls (Figure 4A–D). Control ovaries presented lipid droplets as punctiform spots in the stroma, follicles and corpora lutea; whereas DHEA ovaries were characterized by larger lipid droplets. Oral administration of both carnitine formulations decreased the number and the size of ovarian lipid droplets, highly induced by DHEA, with a more significant effect in DHEA + C2 group. In all, lipid droplets were mainly located in corpora lutea, in parietal granulosa and thecal cells of growing follicles. Numerous isolated big droplets accumulated inside secondary and tertiary follicles in DHEA + C2.

Protein analysis of 17ß-HSD IV revealed an increased expression in DHEA ovaries, respect to controls (Figure 4E,F). Administration of carnitine formulations 1 and 2 reduced the expression of 17ß-HSD IV in comparison to DHEA, with levels lower than control untreated mice.

### 3.2. Oral Administration of Carnitines Formulation 1 and 2 Modulates SIRT1 Functional Network and Mitochondrial Physiology

**Figure 5:**
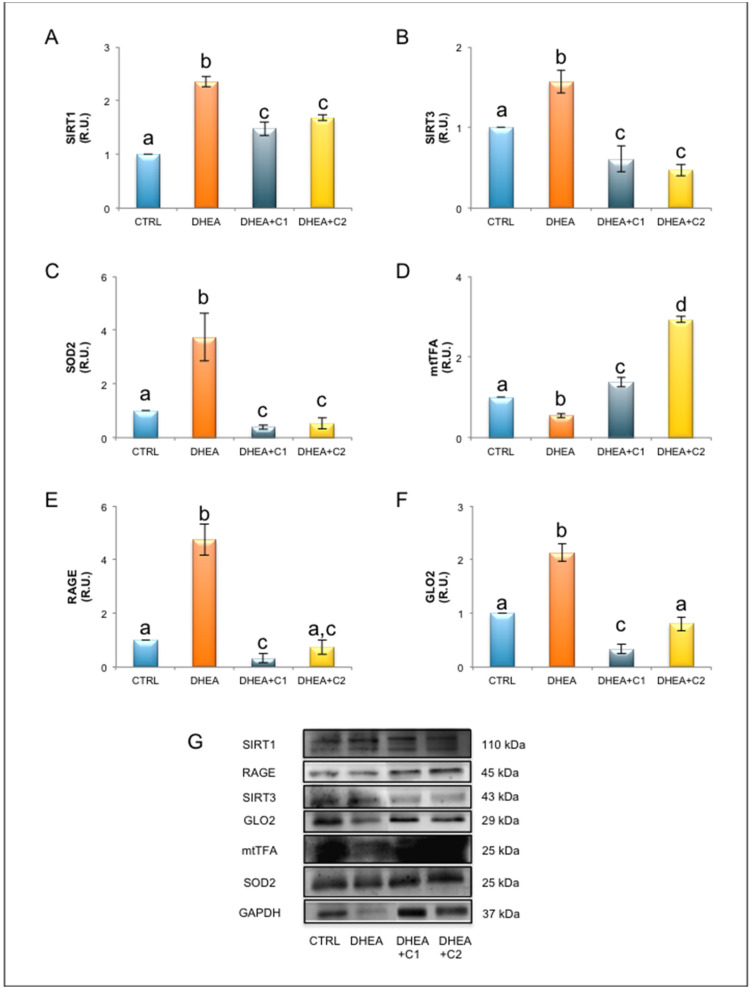
Oral administration of carnitines formulations modulates key elements of SIRT1 functional network, mitochondrial physiology and glycative stress response. Part of the ovaries was processed for protein analysis. Western blot analysis of SIRT1)(**A**), SIRT3 (**B**), SOD2 (**C**), mtTFA (**D**), RAGE (**E**), GLO2 (**F**), and representative images of immunoreactive bands (**G**) are shown. Data are presented as means ± SEM of densitometric analysis of immunoreactive bands normalized to internal reference protein (GAPDH). a, b, c, d *p* < 0.05, *t*-test.

As shown in Figure 5, PCOS induced by DHEA induced a deregulation of SIRT1 functional network involved in antioxidant response. DHEA ovaries presented a significant increase of SIRT1 protein, which was partially prevented by oral administration of carnitine formulation 1 and 2. In addition to SIRT1, we observed in DHEA ovaries increased expression of mitochondrial proteins SIRT3 and SOD2. Administration both carnitine formulations prevented SIRT3 and SOD2 increases in comparison to DHEA, although protein levels were lower than control mice. DHEA administration induced a reduction of mitochondrial mass, as demonstrated by reduced levels of protein expression of mtTFA. By contrast, both carnitine formulations induced an increase of mtTFA, with levels higher than those observed in DHEA and in control ovaries. This effect was more pronounced in ovaries from mice receiving PLC (carnitine formulation 2, DHEA + C2).

### 3.3. Oral Administration of Carnitine Formulation 2 Is Able to Prevent the Establishment of Glycative Stress of DHEA Mice, While Carnitine Formulation 1 Is Not

**Figure 6:**
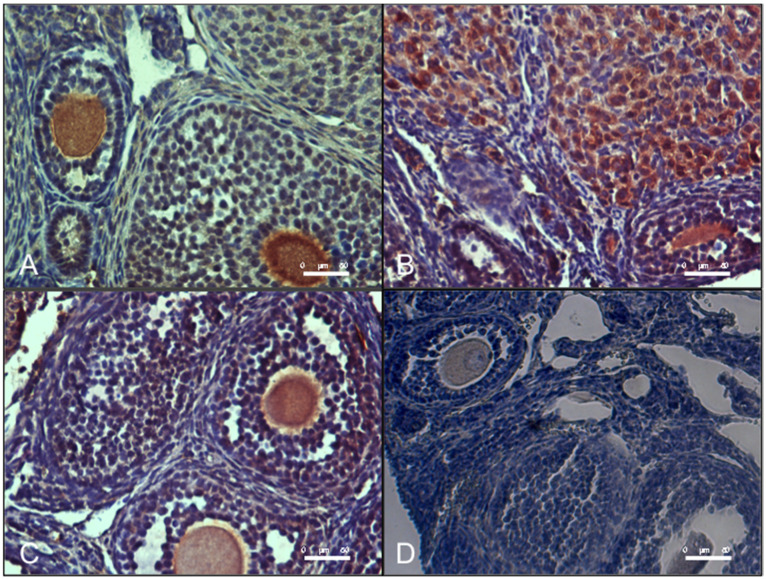
Oral administration of carnitine formulation 2 prevents the accumulation of methylglyoxal-advanced glycation end-products (MG-AGE) induced DHEA. Part of paraffin embedded sections of formalin-fixed ovarian tissue were processed immunohistochemical analysis of MG-AGE accumulation. Representative images of immunolocalization of MG-AGE in CTRL (**A**), DHEA (**B**), DHEA + C1 (**C**) and DHEA + C2 (**D**) ovaries are shown.

As shown in Figure 6, ovaries from DHEA mice presented an increased level of MG-AGE as evaluated by immunohistochemical analysis. Administration of carnitine formulation 2 but not 1 was able to prevent MG-AGE accumulation, with levels similar to controls.

In accordance with this observation, protein analysis of RAGE revealed that DHEA-induced PCOS induced an increase expression of RAGE protein. By contrast, ovaries from mice receiving carnitine formulation 1 and 2 presented lower levels of RAGE in comparison to DHEA (Figure 5). Administration of carnitine formulation 1 induced a reduction of RAGE at levels lower than control, while carnitine formulation 2 ovaries presented RAGE levels similar to controls. When focusing on GLO2, which is the rate-limiting enzyme of the MG detoxification system [34], we observed an increase of GLO2 protein expression in DHEA mice (Figure 5). Administration carnitine formulation 1 and 2 induced prevented GLO2 increase observed in DHEA ovaries. Nevertheless, carnitine formulation 1 presented GLO2 levels lower than control, while the presence of PLC (carnitine formulation 2, DHEA + C2) preserved GLO2 expression at levels similar to control.

## 4. Discussion

In the present study, we have relied on a mouse DHEA model of PCOS to demonstrate that oral administration of acyl-L-carnitines alleviates ovarian dysfunctions associated with this syndrome and to explore whether the co-administration of PLC provides better activity. In order to select the proper protocol for LC and ALC administration we based on studies on male and female fertility [10,14,30,35,36,37,38]. For PLC, since there are no studies on fertility, we employed the minimum effective dose identified by Delaney et al. [39].

Overall, both carnitine formulations tested in this work showed some beneficial effects on functional parameters and counteract oxidative and glycative stress induced by DHEA in the ovarian microenvironment [7]. However, only the formulation containing PLC was effective in maintaining serum testosterone to basal levels in concomitance with major effects on oestrus cycling and mitochondria. This represents the first attempt to investigate therapeutic effects of PLC in PCOS and to characterize the molecular pathways underlying beneficial effects of acyl-L-carnitines at ovarian level.

Regarding LC and ALC, our results are in line with previous observations on humans and mouse model. Numerous studies report that LC supplementation counteracts metabolic and hormonal PCOS symptoms and positively affects folliculogenesis, ovulation and pregnancy rate [4,9,23,24,25,26,27,28,29], possibly in relation to favorable influence on serum biomarkers of oxidative stress [40]. Samimi et al. [10] observed that PCOS patients receiving both ALC and LC presented the reduction of body weight and insulin resistance. With respect to PLC, our observations that PLC addition to the LC-ALC formulation counteracts DHEA-induced androgenism and exhibit greater effectiveness in restoring oestrus cycling represent the first evidence for the potential therapeutic application of PLC in PCOS. Indeed, only 20% of mice receiving LC-ALC formulation cycled, while the addition of PLC resulted in 100% of mice cycling during the treatment, although they were not synchronized. This behavior may be related to carnitine systemic action, including modulation of hypothalamic-pituitary-gonadal (HPG) axis that influences gonadotropin production. It is known that ALC administration significantly modulates GnRH and LH secretion in hypothalamic amenorrhea in hypogonadotropic patients [41]. Studies on specific effects of PLC on HPG will be helpful to clarify mechanism of action of PLC on reproductive functions.

On the other hand, we show that both carnitine formulations tested in this work are able to counteract the effect of DHEA on ovulation rate and oocyte quality, so providing evidence for positive impact of carnitines on folliculogenesis probably related to direct and indirect effects. Indeed, an improved ovarian environment is revealed by the observation of reduced collagen deposition and follicle atresia as well as a restored steroidogenesis as revealed by decreased lipid metabolism and reduced expression of 17ß-HSD IV. Direct effects of carnitines on the cumulus-oocyte complex (COC) may also be hypothesized. LC supports lipid metabolism of COC by transferring fatty acids into the mitochondria and facilitating β-oxidation [42]. Proper energy supply is an essential condition for assuring correct spindle assembly and chromosome separation during meiosis [43]. In this respect fatty acid oxidation represents an important energy source for the oocyte as evidenced by the observation that it is increased by LC exposure of COCs during in vitro maturation and in vitro follicle culture in association with improved oocyte competence [44,45]. A further evidence for the relevance of this mitochondrial energy pathway is the observation that carnitine palmitoyltransferase-II (CPT-II) expression in mouse oocytes has been correlated with oocyte developmental competence. LC uses the Na^+^-driven LC/organic cation transporter-2 (OCTN-2) for its transport into the oocytes, where it is converted to ALC by carnitine palmitoyltransferase-I (CPT-I) in the outer mitochondrial membrane and CPT-II helps in the regeneration of carnitine from acyl-carnitine after the translocation of long chain fatty acids into the mitochondrial matrix [46].

Turning our attention to ovarian mitochondria, the results of this study allow us to hypothesize that these organelles represent possible targets of carnitine activity at ovarian level. In fact, the expression of mtTFA was significantly increased following the administration of carnitines, and a significantly increased effect was observed in the PLC receiving group. Mitochondrial transcription factor A (mtTFA) is a major mitochondrial gene-regulator and acts as a packaging molecule by storing a single copy of mtDNA in a functional mitochondrial nucleoid [47]. Mitochondrial nucleoids contain essential enzymes of an integral antioxidant system, including SOD2 [48] that mitigates oxidative damage and protect mtDNA. Muscle-specific overexpression of mtTFA attenuates the effects of high-fat diet on fat gain and insulin resistance in mice. Beyond its direct effect on mitochondrial DNA replication and transcription, mtTFA overexpression increased Krebs cycle, citrate synthase and fatty acid oxidation in concert with higher β-oxidation capacity [49]. Thus, similar effects by PLC may be hypothesized at ovarian level and investigated in further studies.

Although the synergic effects of LC, ALC, and PLC are strongly supported by present results, the increased therapeutic effects related to PLC supplementation compared with LC and ALC co-administration may be due to its major bioavailability with subsequent enhanced ATP production, superoxide scavenger ability, and protective effects against insulin resistance [20,21,50]. Moreover, from this work emerges a greater effectiveness of the PLC formulation against glycative stress when compared with LC-ALC. In recent years, glycative stress from advanced glycation end products (AGEs) and highly reactive dicarbonyls, such as methylglyoxal, has gained great attention for its putative involvement in PCOS [51,52]. Altered glucose and oxidative stress are considered the major causes of increased formation of AGEs by non-enzymatic glycation in PCOS. AGEs and their receptor RAGEs play an important role in generation of inflammatory molecules and oxidative stress [53]. Here, we show that PLC exerts a powerful anti-glycative activity as supported by the decreased expression of RAGEs and MG-AGE deposition associated with the recovering of basal levels of GLO2, the rate limiting enzyme in the glyoxalase system devoted to MG detoxification, and antiglycative action [34].

Recently, the interest in the involvement of sirtuins in PCOS development and progression has been increasing [54]. In accordance, in our previous work, we found that DHEA mice exhibited enhanced ovarian expression of SIRT1 and SIRT3. Mammals have seven sirtuin proteins, denoted as SIRT1 through SIRT7, each of which regulates diverse physiological processes ranging from metabolism to epigenetic modification by deacetylating numerous substrates in a NAD^+^ dependent manner. SIRT1 is localized to both the nucleus and cytoplasm, whereas SIRT3 is exclusively localized to the mitochondria, and both regulate energy metabolism in response to metabolic stress. Indeed, increased expression of SIRT1 is a marker of mild oxidative stress, which in DHEA ovaries is associated with the rise in protein levels of SIRT3 and SOD2, which are mitochondrial elements of SIRT1 functional network involved in the antioxidant response [7,31]. The effectiveness of both carnitine formulation in preventing upregulation of SIRT1, SIRT3, and SOD2 proteins is a strong indication that carnitine alleviates PCOS altered redox milieu. On the other hand, the finding of differences in the expression of SIRT1, SIRT3, and SOD2 proteins between control and the carnitine formulations may be taken as evidence of a system which has not completely restored homeostatic conditions.

## 5. Conclusions

Although the relevance of the results here obtained is limited by the use of an animal model, our study confirms the hypothesis that oral administration of acyl-L-carnitines alleviates ovarian dysfunctions associated with PCOS and that co-administration of PLC provides better activity. Molecular mechanisms underlying these effects include anti-oxidant/glycative activity and potentiation of mitochondria. In conclusion, although further animal and clinical studies are needed, these molecules show great promise as treatment or co-treatment options in strategies for ameliorating fertility potential in PCOS patients.
